# Isolation, Identification and Insecticidal Activity of the Secondary Metabolites of *Talaromyces purpureogenus* BS5

**DOI:** 10.3390/jof8030288

**Published:** 2022-03-11

**Authors:** Ying Yue, Mingfang Jiang, Hanying Hu, Jinghui Wu, Haoran Sun, Hong Jin, Taiping Hou, Ke Tao

**Affiliations:** Key Laboratory of Bio-Resource and Eco-Environment of Ministry of Education, College of Life Sciences, Sichuan University, Chengdu 610017, China; yueying@stu.scu.edu.cn (Y.Y.); jiangmingfang@stu.scu.edu.cn (M.J.); huhanying@stu.scu.edu.cn (H.H.); wujinghui@stu.scu.edu.cn (J.W.); sunhaoran@stu.scu.edu.cn (H.S.); jinhong@scu.edu.cn (H.J.); houtplab@scu.edu.cn (T.H.)

**Keywords:** *Talaromyces purpureogenus*, *Locusta migratoria manilensis*, Tibetan Plateau, insecticidal activity, locust, UPLC-Q-Exactive Orbitrap/MS

## Abstract

The fungal strain BS5 was isolated from a soil sample collected in the Tibetan Plateau, which displayed good insecticidal activity and was identified as *Talaromyces purpureogenus* based on morphological and molecular analysis. This study aimed to evaluate the insecticidal activity and identify the active compound of the strain BS5 against the locust *Locusta migratoria manilensis*. The insecticidal activity of the fermented broth of BS5 was at 100% after 7 days against locusts. We extracted the fermented broth of BS5 and then evaluated the insecticidal activity of the extracts against locusts. The ethyl acetate extract exhibited promising activity levels with an LC_50_ value of 1077.94 μg/mL and was separated through silica gel column chromatography. The UPLC-Q-Exactive Orbitrap/MS system was employed to analyze the active fraction Fr2.2.2 (with an LC_50_ value of 674.87 μg/mL), and two compounds were identified: phellamurin and rubratoxin B.

## 1. Introduction

As one of the most dangerous agricultural pests, the locust (Orthoptera: Acridoidea) is able to multiply to disastrous levels and form massive swarms that can migrate hundreds of kilometers in one day [1,2]. The invasion of this group of insects is so catastrophic that it affects a great deal of land worldwide, resulting in famine, economic loss and even social disorder [1,2,3]. Consequently, it has become a threat to crop production on an international scale. China, one of the most affected countries, where more than 2 million hectares of agricultural land are damaged by locust invasion annually, has suffered from agricultural and biological disasters [4]. Among all the locust species invading China, the locust *Locusta migratoria manilensis* occupies a dominant position [4]. Chemical insecticides have been commonly used to control agricultural pests. However, the harmful impacts of toxic chemical insecticides have driven extensive research for alternatives, especially biological control agents such as fungus and bacteria [5].

The research and development of microorganisms have shown favorable prospects in the area of biological control. A variety of microorganisms has been isolated from extreme environments, and some potential biocontrol strains and their metabolites have been studied. For example, Djinni et al. [6] isolated a *Streptomyces sundarbansensis* strain from marine brown algae *Fucus* sp. collected from the Algerian coastline that produced bioactive secondary metabolites. Slama et al. [7] reported the isolation of a novel actinomycete strain from northern Tunisian soil that exhibited potent broad-spectrum antibacterial activity. The Tibetan Plateau is the largest and highest plateau in China, with an average altitude higher than 4000 m. This area is also known as the third pole of the world and possesses the third largest continental ice reservoir [8]. The climate of the Tibetan Plateau is unique compared with other alpine and tundra ecosystems [9] and is quite varied due to its huge size, with a geographic range from 30 to 38° N and 70 to 100° E [10]. As a result, the Tibetan Plateau contains an abundant resource of microorganisms, and many promising microbial resources are waiting to be utilized. Additionally, it provides a new path for us to search for microorganisms producing secondary metabolites with novel structures and good activities.

In this study, we isolated microorganisms from soil samples from the Tibetan Plateau. In the course of searching for bioactive natural products from microorganisms to control locust *L. migratoria manilensis*, we found the fungus BS5.

## 2. Materials and Methods

### 2.1. Isolated Strains

The strains were isolated from a soil sample collected in Baingoin County, Tibet, China (31.32° N, 90.04° E). One g of soil sample was collected in 100 mL of sterile distilled water and mixed thoroughly in a shaker for 30 min at 120 rpm. A total of 1 mL of the suspension was serially diluted until a 10^−5^ dilution was obtained. The diluted samples (200 µL) were spread onto the Potato dextrose agar (PDA) supplemented with 100 mg/L streptomycin sulfate. After culturing at 25 °C for 7–14 days, typical strains were selected according to the colonies’ size, shape, and color. The colonies were picked and purified. Plates containing pure cultures were transferred and stored at 4 °C in an agar slant.

### 2.2. Production of Culture Filtrates and Extracts

The strains isolated from soil were subject to fermentation and toxicity bioassays. Fermentations of fungi were conducted in 0.25 L flasks containing 0.1 L of potato dextrose broth (PDB; 200 g/L potato and 20 g/L glucose), incubating for 7 days in a constant-temperature rotary shaker at 25 °C and 140 rpm. After incubation, the culture filtrates were separated from mycelium by filtration, and then the filtrates were filtered through 0.22 µm membranes. The culture filtrates thus obtained were tested immediately for toxicity to locust *L. migratoria manilensis*.

After bioassays with culture filtrates, the culture filtrates of the bioactive strains were extracted three times with equal volume of different solvents viz. petroleum ether, dichloromethane, ethyl acetate and n-butanol, concentrated under reduced pressure and dried. Additionally, the extracts were stored at 4 °C for subsequent bioassays.

### 2.3. Bioassays

Locust *L. migratoria manilensis* used in this study were reared in the Key Laboratory of Bio-Resource and Eco-Environment of Ministry of Education, Sichuan University. The insects were fed fresh ryegrass. The rearing temperature was 28 °C at 70% relative humidity with the photoperiod of 12:12 (light–dark). Insect bioassays were carried out with third-instar larvae of *L. migratoria manilensis*. Additionally, those insects were starved in a 40 cm × 50 cm × 50 cm cage for 24 h before bioassays.

#### 2.3.1. Bioassays with Culture Filtrates

The first was a set of screening bioassays to determine the most bioactive strain. The treatments used were the culture filtrates cultured in PDB, and the controls were treated with PDB. Insecticidal activities were assayed through toxicity bioassays (locust: insect-dipping method; ryegrass: leaf-dipping method) [11]. The third-instar larvae were separately immersed in each test solution for one second. After taking the larvae out, the remaining liquid outside the larvae was gently wiped off with a filter paper. Then, the larvae were placed in the cage (15 cm × 15 cm × 30 cm) and were fed fresh ryegrass (after soaking in the liquid of the same test solution for 3 s and being air-dried). Fifteen third instar-larvae were used for test solution. Three replicates were used in this bioassay. After treatment, insect mortality was recorded every 24 h for 7 days. Locust death from culture filtrates generally occurred within 7 days.

#### 2.3.2. Bioassays with Extracts

The second set of bioassays was used to select the most active extract for subsequent evaluation. All the stored extracts were mixed with Tween 20 at a ratio of 1:1 (*w*/*w*) in distilled water to obtain the solution concentration of 10,000 μg/mL and serially diluted (concentrations of 5000, 2500, 1250 and 625 μg/mL). Distilled water with 1% Tween 20 was used for the control. Insecticidal activities were assayed through toxicity bioassays (locust: insect-dipping method; Ryegrass: leaf-dipping method) [11]. The third-instar larvae were separately immersed in each test solution for one second. After taking the larvae out, the remaining liquid outside the larvae was gently wiped off with a filter paper. Then, the larvae were placed in the cage (15 cm × 15 cm × 30 cm) and were fed fresh ryegrass (after soaking in the liquid of the same test solution for 3 s and being air-dried). Fifteen third-instar larvae were used for treatment with each concentration. Three replicates were used in this bioassay. After treatment, insect mortality was assessed at 24 h intervals until the 6th day. Locust death from extracts generally occurred within 6 days, with no additional mortality beyond 6 days.

#### 2.3.3. Bioassays with Fractions

Based on the insecticidal activities of extracts, the most active extract was purified by the activity-tracking method combined with the separation method of silica gel column chromatography. The third set of bioassays was used to select the active fraction for subsequent evaluation. The method of the toxicity bioassay was the same as above (Section 2.3.2). The most active extract, which was subjected to silica gel column (200–300 mesh, Qingdao Marine Chemical Factory, Qingdao, China), was given four fractions by using a dichloromethane–methanol (25:1, 10:1, 5:1, 3:1, 0:1, *v*/*v*) gradient elution. Additionally, the active fraction Fr.2 was divided into four fractions by using a dichloromethane–methanol (1:0, 50:1, 25:1, 10:1, 3:1, 0:1, *v*/*v*) gradient elution (silica gel, 200–300 mesh, Qingdao Marine Chemical Factory, Qingdao, China). Additionally, the active fraction Fr.2.2 was divided into three fractions by using a petroleum-ether–ethyl-acetate (10:1, 5:1, 3:1,1:1, 0:1, *v*/*v*) gradient elution (silica gel, 300–400 mesh, Qingdao Marine Chemical Factory, Qingdao, China). The UPLC-Q-Exactive Orbitrap/MS system was employed to analyze the active fraction Fr.2.2.2.

### 2.4. Statistical Analysis

The mortality data of all treated groups were corrected by Abbott’s formula [12] and then analyzed with descriptive statistics, one-way analysis of variance (ANOVA) and Duncan’s test [13] using IBM SPSS Statistics 19 [14]. Duncan’s test was performed to compare the mean values at a significance level of *p* < 0.05. All results are expressed as means ± standard error (SE).

The toxicity regression equation, the LC_50_, the 95% confidence interval and the correlation coefficient were calculated using probit analysis [15] by DPS software [16] (probability value analysis was carried out for the data obtained by the bioassays with ethyl acetate extract and fractions).

### 2.5. UPLC-Q-Exactive Orbitrap/MS Analysis

The column was Acquity UPLC CHS C_18_ Column (100 mm × 2.1 mm, 1.7 μm). The mobile system was mobile phase C—acetonitrile (0.1% formic acid) and mobile phase D—0.1% formic acid in water. Gradient elution procedures were: 0–10 min (5–60% C), 10–11 min (60–90% C) and 11–15 min (90% C), at a flow rate of 0.3 mL/min. The column temperature was kept constant at 40 °C, and the injection volume was 5 μL. The specific parameter conditions are shown in Table 1.

**Figure 1:**
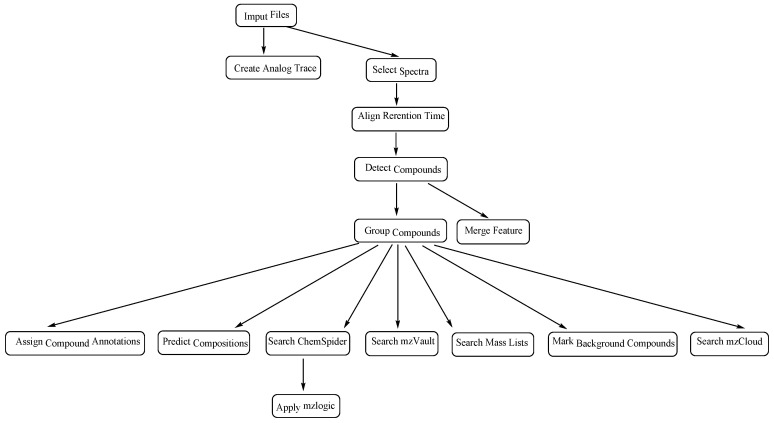
Method route of calculation.

The data acquisition software used was Xcalibur 4.0 (Thermo Fisher Scientific Inc., Waltham, MA, USA), and the data analysis used the Compound Discoverer^TM^ 3.0 software [17]. The sample data file and blank data file were imported into the Compound Discoverer^TM^ software, and the data type was set to “sample” and “blank”, respectively. The unknown calculation method “Untargeted E&L workflow without statistics: Find and identify unknowns” was selected from the algorithm template “E and L Unknown ID with Online and Local Database Searches”. This calculation method performed retention time alignment, unknown compound detection and compound grouping across all samples and predicted elemental compositions for all compounds. We identified compounds using mzCloud (ddMS^2^), ChemSpider (exact mass or formula) and local database searches against mass lists (exact mass and RT) and mzVault spectral libraries, performed similarity search for all compounds with ddMS^2^ data using mzCloud and applied mzLogic to rank order structures from ChemSpider and mass list search results. The calculation method route is shown in Figure 1.

### 2.6. Fungal Identification

#### 2.6.1. Morphological Analysis

Cultures were inoculated in three-point fashion onto MEA (Malt Extract Agar; glucose 20 g/L, tryptone 1 g/L, maltose 20 g/L, agar 20 g/L), OA (Oatmeal Agar; oatmeal 20 g/L, FeSO_4_·7H_2_O 0.001 g/L, ZnSO_4_·7H_2_O 0.001 g/L, agar 18 g/L, MnCl_2_·4H_2_O 0.001 g/L), YES (Yeast Extract Sucrose Agar; yeast extract 20 g/L, sucrose 150 g/L, MgSO_4_·7H_2_O 0.5 g/L, ZnSO_4_·7H_2_O 0.01 g/L, CuSO_4_·5H_2_O 0.005 g/L, agar 20 g/L), DG18 (Dichloran 18% Glycerol Agar; tryptone 5 g/L, glucose 10 g/L, chlornitramine 0.002 g/L, KH_2_PO_4_ 1 g/L, glycerol 200 g/L, MgSO_4_·7H_2_O 0.5 g/L, chloramphenicol 0.1 g/L, agar 15 g/L, pH 5.6 ± 0.2), CYA (Czapek Yeast Autolysate; sucrose 30 g/L, yeast extract 5 g/L, NaNO_3_ 3 g/L, KCl 0.5 g/L, MgSO_4_·7H_2_O 0.5 g/L, FeSO_4_·7H_2_O 0.01 g/L, ZnSO_4_·7H_2_O 0.01 g/L, CuSO_4_·5H_2_O 0.005 g/L, K_2_HPO_4_ 1 g/L; agar 20 g/L) and CREA (Creatine Sucrose Agar; sarcosine 3 g/L, sucrose 30 g/L, KCl 0.5 g/L, MgSO_4_·7H_2_O 0.5 g/L, agar 20 g/L, FeSO_4_·7H_2_O 0.01 g/L, K_2_HPO_4_ 1.3 g/L, bromocresol purple 0.05 g/L) using 90 mm Petri dishes. Plates were incubated for 7–14 days at 25 °C in darkness.

Mycelium images were observed with the DM4B fluorescence microscope (Leica, Wetzlar, Germany) using colonies grown on CYA after 14 days. Morphological characters were analyzed using a Hitachi SU3500 scanning electron microscope (SEM).

#### 2.6.2. Molecular Identification and Phylogenetic Analysis

Fungus BS5 was grown on PDA and incubated at 25 °C for 7 days before harvesting for DNA extraction. The total genomic DNA was extracted using the CTAB method [18]. The DNA was assessed by 0.8% agarose gel electrophoresis. The ITS sequence of fungus BS5 was amplified using the universal primers ITS1 (5′-TCCGTAGGTGAACCTGCGG-3′) and ITS4 (5′-TCCTCCGCTTATTGATATGC-3′) [19]. A fragment of the β-tubulin gene was amplified using the primers bt2a (5′-GGTAACCAAATCGGTGCTGCTTTC-3′) and bt2b (5′-ACCCTCAGTGTA GTGACCCTTGGC-3′) [20]. Polymerase Chain Reaction (PCR) amplification was performed on the Bio-Rad S1000 thermal cycler (Bio-Rad, Hercules, CA, USA), using a total of 25 μL of reaction, including 12.5 μL of 2× Taq PCR Master Mix (Tiangen Biotech, Beijing, China), 0.5 μL of each forward and reverse primer, 1 μL of DNA template and 10.5 μL of double-sterilized distilled water (ddH_2_O). The amplification program included an initial denaturation at 94 °C for 5 min, followed by 35 cycles of denaturation at 94 °C for 30 s, annealing at 55 °C for 30 s and extension at 72 °C for 30 s. Finally, an extension at 72 °C for 10 min was conducted. Following PCR, the PCR product was sequenced by Beijing TsingKe Biological Technology Company (Kunming, Yunnan, China), and the obtained sequences were submitted to GenBank and compared using the National Center for Biotechnology Information database [21]. A phylogenetic tree was constructed using the Maximum Likelihood method and the MEGA 7.0 program (Molecular Evolutionary Genetics Analysis, http://www.megasoftware.net (accessed on 3 February 2021)) with bootstrap values based on 1000 replicates.

## 3. Results

### 3.1. Determination of Insecticidal Activity of Culture Filtrates

We conducted preliminary bioassays with 28 fungal culture filtrates. Among them, the culture filtrates of BS3, BS4, BS5 and HS1 showed more than 50% mortality within 7 days, and the culture filtrate of BS5 showed the highest mortality rate (100%) against third-instar larvae of *L. migratoria manilensis*.

### 3.2. Determination of Insecticidal Activity of Extracts and Fractions of Fungus BS5

The four extracts of fungus BS5 screened in this study were tested for the bioassays against third-instar larvae of *L. migratoria manilensis*. At concentrations of 5000 and 10,000 µg/mL, the ethyl acetate extract showed the highest mortality rate (over 80%), while the other extracts exhibited much lower insecticidal activity (Table 2).

Ethyl acetate extract displayed good insecticidal activity toward locusts with an LC_50_ value of 1077.94 μg/mL (Table 3). For further work based on this result, ethyl acetate extract was selected. The fractions separated from the ethyl acetate extract were evaluated for their insecticidal activity against third-instar larvae of *L. migratoria manilensis* (Table 3). The ethyl acetate extract was given four fractions (Fr.1–Fr.4), and Fr.2 was the most active fraction, with an LC_50_ value of 896.66 µg/mL. Then, the active fraction Fr.2 was given four fractions (Fr.2.1–Fr.2.4), and fraction Fr.2.2 displayed the best insecticidal activity with an LC_50_ value of 462.89 µg/mL, while fraction Fr.2.1, Fr.2.3 and Fr.2.4 were inactive. Next, the active fraction Fr.2.2 was given three fractions (Fr.2.2.1–Fr.2.2.3), and the LC_50_ values of Fr.2.2.1 and Fr.2.2.2 were 1278.95 and 674.87 µg/mL, respectively. Fraction Fr.2.2.3 was not evaluated for insecticidal activity because of the shortage in weight.

### 3.3. UPLC-Q-Exactive Orbitrap/MS Analysis of Fr.2.2.2

**Figure 2:**
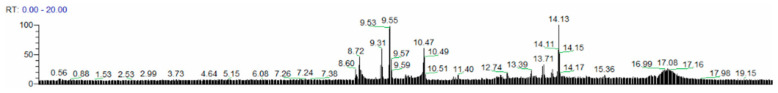
Ion flow chromatogram of Fr.2.2.2.

**Figure 3:**
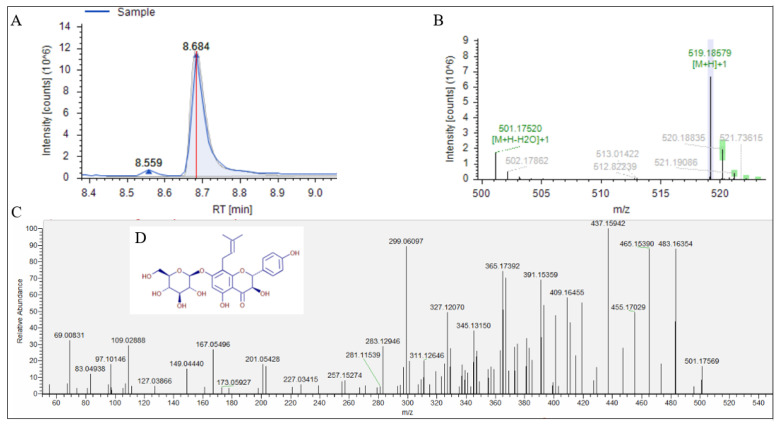
Analysis of No.1. (**A**) chromatogram of No.1. (**B**) parent ion and isotope. (**C**) The MS^2^ spectrum of parent ion *m*/*z* 519.18579. (**D**) Structure of phellamurin.

**Figure 4:**
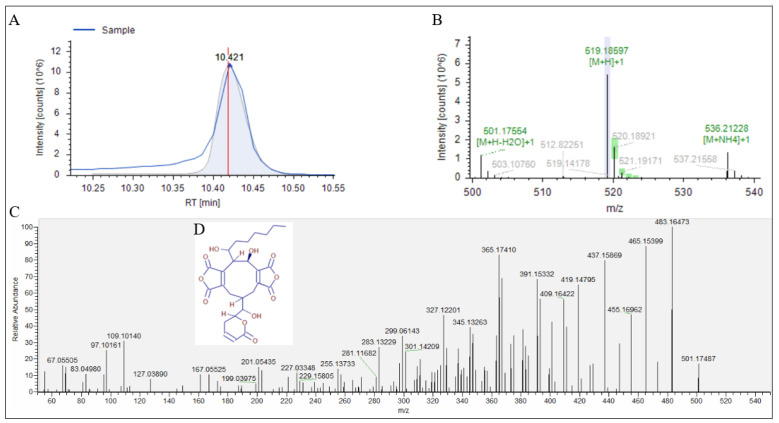
Analysis of No.2. (**A**) chromatogram of No.2. (**B**) parent ion and isotope. (**C**) The MS^2^ spectrum of parent ion *m*/*z* 519.18597. (**D**) structure of rubratoxin B.

We used UPLC-Q-Exactive Orbitrap/MS to further analyze Fr.2.2.2, and its total ion flow chromatogram is shown in Figure 2. The compounds in the Fr.2.2.2 were separated, showing multiple peaks. The obtained data file and the blank data file were imported into the Compound Discoverer^TM^ software for calculation. All calculation results were compared with the local and online databases, and the parent ions, isotopes and secondary ion fragments of the only two compounds, their structures being phellamurin (Figure 3) and rubratoxin B (Figure 4), respectively, could match the compounds listed in the database (Table 4).

### 3.4. Fungal Identification of BS5

The strain BS5 had been identified as the genus *Talaromyces* (Eurotiomycetes, Trichocomaceae) in terms of molecular identification and phylogenetic analysis, and this was confirmed by examining its morphology with reference to Samson et al. [22] and Yilmaz et al. [23]. The genus *Talaromyces* was initially designated to the *Penicillium* species. However, the concept of *Talaromyces* has been lately extended to include all species in the *Penicillium* subgenus *Biverticillium* following the “one fungus—one name” principle confirmed recently in fungal taxonomy [22,23]. Thus, the fungi *Talaromyces purpureogenus*, which was previously classified as the *Penicillium* subgenus *Biverticillium*, now belongs to *Talaromyces*.

#### 3.4.1. Morphological Analysis

**Figure 5:**
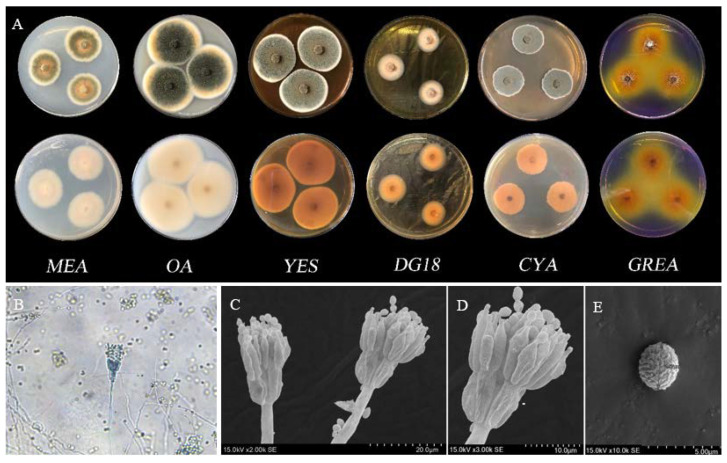
Morphological characters of *Talaromyces purpureogenus* BS5. (**A**) Colonies from left to right as follows: (top row) MEA, OA, YES, DG18, CYA and CREA obverse view; (bottom row) MEA, OA, YES, DG18, CYA and CREA reverse view. (**B**) Diagram of OM (Optical Microscope). (**C**–**E**) Diagram of SEM (Scanning Electron Microscope).

For morphological identification, micro and macromorphology analyses were performed as described in Samson et al. and Yilmaz et al. [22,23]. The morphological characteristics of strain BS5 are shown in Figure 5. On CYA, 25 °C, 7 days: colony diam 26–28 mm, mycelia white, texture floccose; sporulation dense, conidia en masse grayish green; reverse brownish orange. On MEA, 25 °C, 7 days: colony diam 32–34 mm, mycelia white and light orange, texture floccose; sporulation moderately dense, conidia en masse dull green; reverse white. On YES, 25 °C, 7 days: colony diam 42–44 mm, mycelia white, texture floccose; sporulation moderately dense, conidia *en masse* dull green to greyish green; reverse brownish orange. On DG18, 25 °C, 7 days: colony diam 23–25 mm, mycelia white and bright orange, texture velvety and floccose; sporulation sparse, conidia en masse dull green; reverse light orange. On OA, 25 °C, 7 days: colony diam 48–55 mm, mycelia white and light orange, texture floccose; sporulation moderately dense, conidia en masse dark green; reverse orangish white. On CREA, 25 °C, 7 days: colony diam 24–26 mm, acid production. The main morphological characteristics are shown as the connected spherical conidia at the top of the conidiophore’s broom-like branches.

#### 3.4.2. Molecular Identification and Phylogenetic Analysis

**Figure 6:**
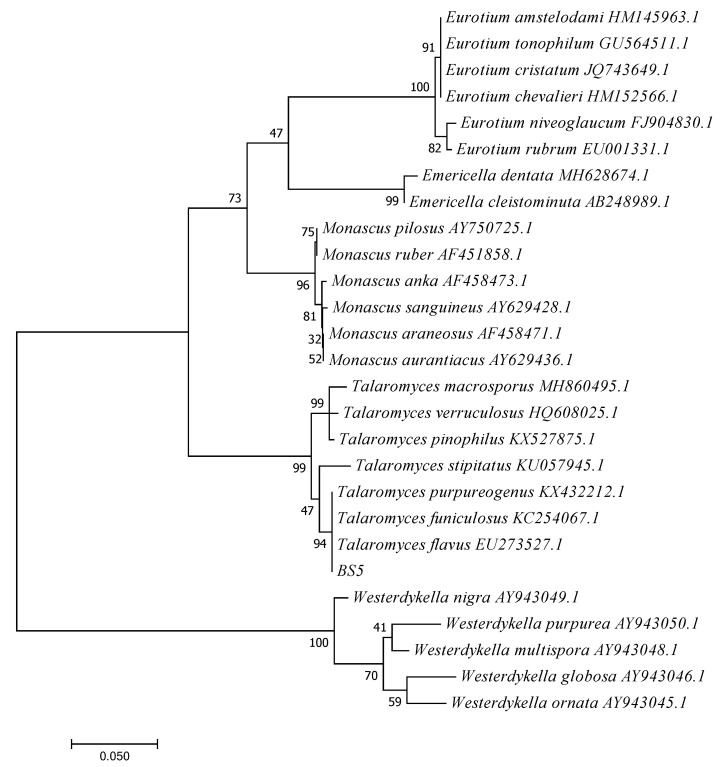
The Maximum Likelihood method phylogram yielded from ITS sequence data set.

The molecular analysis of the ITS region of BS5 showed its high similarity to the species *Talaromyces* *purpureogenus* (99%), *Talaromyces funiculosus* (99%) and *Talaromyces flavus* (99%) among the nucleotide sequences available in the NCBI database. The online blast result indicates that the sequences of fungus BS5 were a 99% match for the genus *Talaromyces*. Figure 6 presents the phylogenetic reconstruction of the genera *Talaromyces*, *Eurotium*, *Emericella*, *Monascus* and *Westerdykella*. Additionally, we could conclude that fungus BS5 belongs to the genus *Talaromyces* sp. (MH071393).

**Figure 7:**
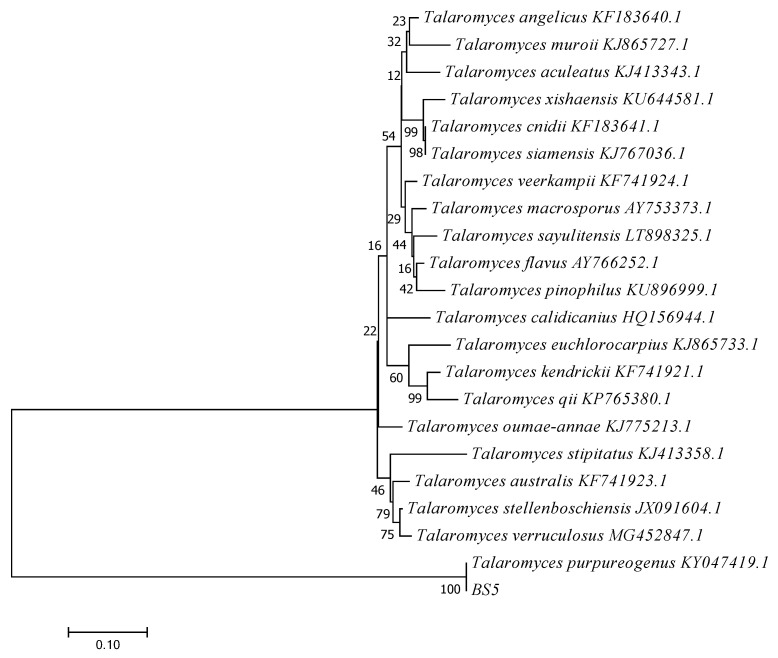
The Maximum Likelihood method phylogram yielded from partial β-tubulin sequence data set.

Then, a fragment of the β-tubulin gene of the BS5 strain was amplified to obtain to the species determination. The molecular analysis of the β-tubulin region of BS5 showed its high similarity to the species *Talaromyces purpureogenus* (100%) among the nucleotide sequences available in the NCBI database (Figure 7). Fungus BS5 belonged to *Talaromyces purpureogenus* (MK327544).

## 4. Discussion

The Tibetan Plateau, which has a unique geographical location, special climatic conditions and a natural environment and ecosystem, contains an abundance of microorganisms. Additionally, it provides a new path for us to search for microorganisms producing secondary metabolites with novel structures and good activities. In our study, 28 strains were isolated from a soil sample collected in Tibet, and the fermented broth of fungus BS5 showed the highest mortality rate (100%) after 7 days against third-instar larvae of *L. migratoria manilensis*. Based on the morphological and molecular identification, the fungus BS5 was identified as *T. purpureogenus*.

The genus *Talaromyces* (Eurotiomycetes, Trichocomaceae) is an important species due to its widespread use in food, agricultural production and medicine. At present, some strains and their metabolites have been studied, and many secondary metabolites have shown good biological activity, such as antibacterial [24,25,26], antitumor [27,28,29], antiviral [30,31] and enzyme inhibition [32,33] activity. In addition, researchers reported its application in insect biocontrol, such as silkworms [34,35] and notorious nematodes [36]. To date, few studies have investigated its insecticidal activity against locust *L. migratoria manilensis*. Therefore, the potential of these fungi must be explored. Additionally, our study indicated that the fermented broth of fungus BS5 *T. purpureogenus* had insecticidal activity against locust *L. migratoria manilensis*. The petroleum ether, dichloromethane, ethyl acetate and n-butanol extracts of the fermented broth of fungus BS5 were tested for bioassays against the locust *L. migratoria manilensis*. Among them, the ethyl acetate extract showed the strongest insecticidal activity. At concentrations of 5000 and 10,000 µg/mL, the ethyl acetate extract showed the highest mortality rate (over 80%), while the other extracts exhibited much lower insecticidal activity. Ethyl acetate extract displayed good insecticidal activity with an LC_50_ value of 1077.94 μg/mL after 6 days. The ethyl acetate extract was therefore fractionated by silica gel column chromatography to isolate and purify its bioactive chemical constituents.

Phellamurin is a flavonoid glycoside that is abundant in the leaves of Phellodendron wilsonii Hayata et Kanehira (Rutaceae). Studies have reported its various biological activities, such as antitumor function. Additionally, Honda et al. [37] reported that phellamurin can inhibit egg laying by *Papilio protenor*. However, few studies showed its insecticidal activity against locusts. Structurally, rubratoxins belong to the family of nonadrides, a group of fungal natural products with a cyclononane ring and two fused maleic anhydrides to form the 5–9–5 core ring system [38]. *T. purpureogenus* can produce extracellular enzymes and red pigment and also produces mycotoxins, such as rubratoxin A and B [39,40]. Samson et al. [41] reported that *Talaromyces* and *Penicillium* can produce rubratoxins. Wang et al. [42] reported that metabolite rubratoxin B yielded from a bioassay-guided fractionation of soil fungus *Penicillium purpurogenum* fermentation using a morphological deformation of *Pyricularia oryzae* mycelia exhibited cell toxicity, inhibition of cell proliferation and matrix metalloproteinase 2 and 9 activities. The literature has demonstrated the presence of rubratoxins in *T. purpureogenus*. At present, there are a large number of studies on the biological activity of rubratoxin B with antitumor and bacteriostatic activity. However, few studies showed insecticidal activity. In our study, the separated fractions from the ethyl acetate extract were evaluated for their insecticidal activity against third-instar larva of *L. migratoria manilensis*. Additionally, fraction Fr.2.2.2 displayed good insecticidal activity with an LC_50_ value of 674.87 μg/mL, and we used UPLC-Q-Exactive Orbitrap/MS for further analysis. The results show phellamurin and rubratoxin B were in fraction Fr.2.2.2, but the insecticidal activity of the compounds needs to be further investigated. In addition, further research is also needed to evaluate the mechanism of insecticidal activity.

## 5. Conclusions

In this report, fungus *T. purpureogenus* BS5, which was isolated from a soil sample collected in the Tibetan Plateau, was found to be insecticidally active against locust *L. migratoria manilensis*, which had a certain reference value for the biological control of locust. Furthermore, fraction Fr.2.2.2 displayed good insecticidal activity against locusts. The results show phellamurin and rubratoxin B were in fraction Fr.2.2.2, and the results may serve as a screening medium for a leader compound in the development of insecticidal drugs.

## Figures and Tables

**Table 1:** Parameter setting of UPLC-Q-Exactive Orbitrap.

	Parameter	Set Value
Source of ion	Spray voltage	3.5 kV (+)/3.2 kV (−)
	Capillary temperature	320 °C
	Sheath gas	35 arb
	AUX gas	10 arb
	Sweep gas	0 arb
	Probe heater temperature	350 °C
	S-lens	60
Mass spectrometry scanning	Scan mode	Full MS-ddms^2^
	Full MS scan range	100 to 1500 *m*/*z*
	Spectrum data type	Profile
	Resolution	Full MS: 70,000
		MS/MS: 17,500
	AGC target	Full MS: 1e^6^
		MS/MS: 2e^5^
	Maximum IT	Full MS: 100 ms
		MS/MS: 50 ms
	Loop count	3
	MSX count	1
	Isolation width	1.5 *m*/*z*
	NCE (stepped NCE)	20, 40, 60
	Minimum AGC target	8e^3^
	Intensity threshold	1.6e^5^
	Dynamic exclution	5 s

**Table 2:** The mortality rates of extracts of fungus BS5.

Extracts	Concentration	Mortality (%; means ± SE)			
	(µg/mL)	1st Day	2nd Day	3rd Day	4th Day
PEE	625	0.00 ± 0.00	0.00 ± 0.00	0.00 ± 2.27	0.00 ± 4.65h
	1250	0.00 ± 0.00	0.00 ± 0.00	0.00 ± 2.27	0.00 ± 4.65h
	2500	0.00 ± 0.00	0.00 ± 0.00	0.00 ± 2.27	0.00 ± 4.65h
	5000	0.00 ± 0.00	0.00 ± 0.00	0.00 ± 2.27	2.33 ± 4.03h
	10,000	0.00 ± 0.00	0.00 ± 0.00	0.00 ± 2.27	6.98 ± 2.33gh
DE	625	0.00 ± 0.00	0.00 ± 0.00	0.00 ± 2.27	0.00 ± 4.65h
	1250	0.00 ± 0.00	0.00 ± 0.00	0.00 ± 2.27	2.33 ± 4.03h
	2500	0.00 ± 0.00	0.00 ± 0.00	2.28 ± 2.27	4.66 ± 4.65h
	5000	0.00 ± 0.00	0.00 ± 0.00	4.55 ± 3.94	11.63 ± 2.32gh
	10,000	0.00 ± 0.00	6.67 ± 3.85	13.64 ± 2.27	27.91 ± 6.15ef
EAE	625	0.00 ± 0.00	8.89 ± 2.22	13.64 ± 2.27	37.21 ± 4.03e
	1250	0.00 ± 0.00	11.11 ± 2.22	25.00 ± 3.94	55.82 ± 4.65cd
	2500	0.00 ± 0.00	20.0 ± 3.85	38.64 ± 3.94	67.44 ± 4.65c
	5000	4.45 ± 2.22	24.4 ± 5.88	52.27 ± 3.93	81.40 ± 4.65b
	10,000	17.78 ± 2.22	53.3 ± 3.85	77.27 ± 6.01	93.02 ± 0.00a
NE	625	0.00 ± 0.00	4.45 ± 2.22	6.82 ± 2.27	11.63 ± 2.32gh
	1250	0.00 ± 0.00	6.67 ± 3.85	11.37 ± 3.93	18.61 ± 2.33fg
	2500	0.00 ± 0.00	13.33 ± 3.85	25.00 ± 3.94	34.89 ± 2.32e
	5000	2.22 ± 2.22	15.55 ± 2.22	31.82 ± 3.94	53.49 ± 2.32d
	10,000	6.67 ± 3.85	26.67 ± 6.67	47.73 ± 6.01	60.47 ± 6.15cd

Note: PEE (petroleum ether extract); DE (dichloromethane extract); EAE (ethyl acetate extract); NE (n-butanol extract). Different letters (a–h) in the same column indicate significant differences at the level of *p* < 0.05 (Duncan’s test). Mortality was corrected using Abbott’s formula.

**Table 3:** Toxicity regression equation of ethyl acetate extract and fractions on day 6.

Extract/Factions	TRE *	LC_50_ (µg/mL) (95% CL) ^†^	r ^#^	*p* Value
EAE	y = 0.6140 + 1.4463x	1077.94 (927.46–1252.83)	0.9948	0.0005
Fr.1	y = 1.2406 + 0.9305x	10,968.79 (9340.81–12,880.50)	0.9968	0.0002
Fr.2	y = 0.6119 + 1.4862x	896.66 (576.55–1394.51)	0.9642	0.0081
Fr.3	y = 0.4328 + 1.6338x	2114.72 (1745.95–2561.37)	0.9858	0.0020
Fr.4	y = 1.8511 + 1.6674x	12,845.69 (6790.08–24,301.87)	0.9591	0.0099
Fr.2.1	y = 0.2814 + 1.3418x	8630.19 (6948.45–10,718.98)	0.9927	0.0007
Fr.2.2	y = 2.5809 + 0.9076x	462.89 (389.67–549.86)	0.9970	0.0002
Fr.2.3	y = 2.7254 + 0.6768x	2294.07 (1742.27–3020.64)	0.9708	0.0060
Fr.2.4	y = 1.4135 + 0.8991x	9746.56 (7192.64–13,207.32)	0.9874	0.0017
Fr.2.2.1	y = 2.9517 + 0.6593x	1278.95 (1026.05–1594.17)	0.9868	0.0018
Fr.2.2.2	y = 3.3508 + 0.5829x	674.87 (494.19–921.62)	0.9861	0.0020

Note: * Toxicity regression equation. ^†^ 95% confidence interval. ^#^ Correlation coefficient. LC_50_ (lethal concentration that kills 50% of the larvae); EAE (ethyl acetate extract).

**Table 4:** Chromatography and mass spectrometry information in the components of Fr.2.2.2.

No.	RT (min)	Deduced Compound	Formula	Molecular Weight	Fragment Ions (*m*/*z*)
1	8.684	Phellamurin	C_26_H_30_O_11_	518.17839	365.27392, 437.15942 465.15390, 483.16354
2	10.421	Rubratoxin B	C_26_H_30_O_11_	518.17843	365.17410, 437.15942, 465.15399, 483.16473
